# Physicians using ultrasound in Danish emergency departments are mostly summoned specialists

**DOI:** 10.1186/1757-7241-23-S1-A44

**Published:** 2015-07-16

**Authors:** Klaus Nielsen, Johnny RM Lauridsen, Christian B Laursen, Mikkel Brabrand

**Affiliations:** 1 Department of Respiratory Medicine, Gentofte Hospital, Gentofte, Denmark; 2 Department of Anaesthesia and Intensive Care, Holbæk Hospital, Holbæk, Denmark; 3 Department of Respiratory Medicine, OUH Odense University Hospital, Odense, Denmark; 4 Department of Emergency Medicine, Sydvestjysk Sygehus, Esbjerg, Denmark

## Background

Emergency Ultrasound is a relatively new diagnostic discipline. It is used as an extension of the clinical examination and is ideal in the setting of acute illness. The objective of this study was to investigate how many Emergency Departments (ED) in Denmark have implemented emergency ultrasound. We also wanted to give an idea of how many and which physicians have adopted ultrasound as a diagnostic tool so far.

## Methods

The study was a cross-sectional, descriptive, multicenter survey that included all physician staffed EDs in Denmark. An Internet based questionnaire was distributed by e-mail to all heads of department. Those departments who responded that ultrasound was available in their department were included in the second part of the study where all physicians working in the ED were contacted and asked to complete a second questionnaire.

## Results

All 28 eligible Emergency Departments participated in the first part of the study (Response Rate (RR): 100%). 25 EDs (89%, 95% CI: 85-93) had ultrasound equipment available. Questionnaires were distributed to 1,872 physicians in these departments and 561 responded (RR: 30%, 95% CI: 28-32). Overall 257 (46%, 95% CI: 42-50) were users of Emergency Ultrasound and 304 were non-users (54%, 95% CI: 50-58). The largest group with 146 respondents (25%, 95% CI: 21-29) were anaesthetists with merely consult duty in the ED. When looking exclusively on physicians with on-call duty in the ED, thus excluding anaesthetists, only 146 (35%, 95% CI: 30-40) were users of ultrasound while 269 (65%, 95% CI: 60-70) were non-users. There was a considerable difference regarding age, level of training, and medical specialty between users and non-users. Users were mainly anaesthetists and attending physicians from other departments. The majority of non-users were young physicians with on-call duty in the ED.

## Conclusion

We have found that although almost all Danish EDs have ultrasound equipment available, few ED physicians seem to have adopted the tool. Emergency Ultrasound is mainly performed by specialists who are summoned to the ED in case of severe acute illness and not by those physicians who comprise the backbone of the ED around the clock.

